# A Comparison of Differential Item Functioning Detection Methods in Cognitive Diagnostic Models

**DOI:** 10.3389/fpsyg.2019.01137

**Published:** 2019-05-17

**Authors:** Yanlou Liu, Hao Yin, Tao Xin, Laicheng Shao, Lu Yuan

**Affiliations:** ^1^China Academy of Big Data for Education, Qufu Normal University, Qufu, China; ^2^Department of Psychology, School of Education, Qufu Normal University, Qufu, China; ^3^Collaborative Innovation Center of Assessment toward Basic Education Quality, Beijing Normal University, Beijing, China; ^4^School of Economics and Management, Taishan University, Tai'an, China

**Keywords:** cognitive diagnostic model, Wald statistics, differential item functioning, information matrix, logistic regression method

## Abstract

As a class of discrete latent variable models, cognitive diagnostic models have been widely researched in education, psychology, and many other disciplines. Detecting and eliminating differential item functioning (DIF) items from cognitive diagnostic tests is of great importance for test fairness and validity. A Monte Carlo study with varying manipulated factors was carried out to investigate the performance of the Mantel-Haenszel (MH), logistic regression (LR), and Wald tests based on item-wise information, cross-product information, observed information, and sandwich-type covariance matrices (denoted by *W*_d_, *W*_XPD_, *W*_Obs_, and *W*_Sw_, respectively) for DIF detection. The results showed that (1) the *W*_XPD_ and LR methods had the best performance in controlling Type I error rates among the six methods investigated in this study and (2) under the uniform DIF condition, when the item quality was high or medium, the power of *W*_XPD_, *W*_Obs_, and *W*_Sw_ was comparable with or superior to that of MH and LR, but when the item quality was low, *W*_XPD_, *W*_Obs_, and *W*_Sw_ were less powerful than MH and LR. Under the non-uniform DIF condition, the power of *W*_XPD_, *W*_Obs_, and *W*_Sw_ was comparable with or higher than that of LR.

## Introduction

Cognitive diagnostic models (CDMs) as a class of discrete latent variable models have been developed to provide finer-grained and multidimensional diagnostic feedback information about examinees' strengths and weaknesses on a set of attributes. However, inferences based on CDMs are invalid when an item functions unequally for examinees with the same attribute mastery pattern but from different population groups. In CDMs, an item is assumed to function differently when subjects from different groups but with the same attribute mastery pattern nevertheless have different probabilities of answering the item correctly. Manifest group characteristics (e.g., gender, age, and race/ethnicity) are typically treated as proxy variables that may lead to DIF, and several studies tried to explore underlying sociological and psychological reasons why DIF occurred in practice (see e.g., Svetina et al., 2017; George and Robitzsch, 2018). The occurrence of differential item functioning (DIF) in CDMs could possibly lead to severe consequences, such as inaccurate and imprecise item and attribute mastery pattern estimates (Hou et al., 2014). Given that, great importance has been attached to detecting and eliminating DIF items from cognitive diagnostic tests, DIF should be routinely detected to ensure the fairness and validity of the tests in practice applications.

Several DIF detection methods have been proposed and investigated in the framework of CDMs (Zhang, 2006; Li, 2008; Hou et al., 2014; Wang et al., 2014; Li and Wang, 2015; Liu et al., 2016b), which can be classified into two types, CDM based and not. For example, the modified higher-order DINA (Li, 2008) and log-linear cognitive diagnosis models for DIF assessment (Li and Wang, 2015), for which the model parameters were estimated using the Markov chain Monte Carlo (MCMC) algorithm, and the Wald test, for which MCMC or maximum likelihood estimation was used for estimating the item parameters (Hou et al., 2014; Li and Wang, 2015) are the CDM-based method. The Mantel-Haenszel (MH; Mantel and Haenszel, 1959; Mantel, 1963) test, the simultaneous item bias test (SIBTEST; Shealy and Stout, 1993), and logistic regression (LR; Swaminathan and Rogers, 1990) are non-parametric methods that are not based on CDMs. Zhang (2006) investigated the performance of MH and SIBTEST for DIF detection using attribute mastery profiles as the matching variables; however, the attribute mastery profiles were estimated under the assumption that the item parameters for the reference and focal groups were the same, and both tests exhibited very low power to detect non-uniform DIF. Although the modified higher-order DINA model for DIF analysis proposed by Li (2008) had acceptable Type I error rate control, one of the limitations of Li's method was that the author imposed a very strong assumption on the attribute mastery patterns. Hou et al. (2014) proposed that the Wald statistic can be used to detect DIF, and they found that the performance of the Wald test was comparable with or superior to that of MH and SIBTEST in detecting uniform DIF. However, Hou et al. (2014) and Wang et al. (2014) found that the Wald statistic (*W*_d_) based on the information matrix estimation method developed by de la Torre (2011) yielded inflated Type I error rates. Li and Wang (2015) compared the empirical performance of the LCDM-DIF method with the Wald method for two and three groups using the MCMC algorithm, and they found that the Type I error rates of the LCDM-DIF were better controlled than the Wald statistic under most conditions, however, for the three-group conditions, the power of the Wald method was slightly better than that of the LCDM-DIF. Svetina et al. (2018) evaluated the impact of Q-matrix misspecification on the performance of LR, MH, and *W*_d_ for detecting DIF in CDMs. They found that the Type I error rate control of LR and MH was better than that of *W*_d_; LR and *W*_d_ had greater power than MH and the performance of LR, MH, and *W*_d_ was affected by Q-matrix misspecification.

The *W*_d_ test for DIF detection that was used in previous studies (Hou et al., 2014; Svetina et al., 2018) was based on item-wise information matrix. However, Liu et al. (2016a) pointed out that because the item and the structural model parameters are simultaneously estimated from the item response data, the information matrix of CDMs should contain both item and structural parameters. The item-wise information matrix underestimate the variance-covariance matrix of item parameters (Philipp et al., 2018). As alternatives, cross-product (XPD) information, observed (Obs) information, and sandwich-type (Sw) covariance matrices have been proposed for estimating asymptotic covariance matrices of item parameters (Liu et al., 2016b, 2019; Philipp et al., 2018). That is, the item parameter covariance matrix used to compute the Wald statistic can be estimated using XPD, Obs, or Sw matrix (statistics denoted as *W*_XPD_, *W*_Obs_, and *W*_Sw_, respectively). Liu et al. (2016b) evaluated the empirical performance of *W*_XPD_ and *W*_Obs_ for detecting DIF in CDMs following Hou et al.'s (2014) simulation design. They found that when the sample size was 1,000 and the attribute correlation was 0, following Bradley's (1978) liberal criteria (1978), *W*_XPD_ and *W*_Obs_ had accurate Type I error rates.

In summary, the main focus of this study was to investigate the empirical behavior of the Wald statistic based on the XPD, Obs, Sw, and item-wise information matrices for DIF detection and to compare these Wald statistics with MH and LR using DINA model as an example. The remainder of this article is organized as follows. Firstly, we introduce the DINA model and item parameter covariance matrix estimation procedures as the basis of the estimation of the Wald statistics for DIF detection. Secondly, we outline the DIF detection methods investigated in this study. Thirdly, we present the results of simulation studies conducted to systematically evaluate the DIF detection methods under various conditions. Finally, a discussion of the findings is provided.

## Background

Assume that there are *N* examinees responding to *J* dichotomous items, in which *K* binary attributes are diagnosed. The number of the possible attribute mastery patterns (α1′,···,α1′,···,αL′)′ is *L* = 2^*K*^, ${\text{α}}_{l}={\left(\alpha_{l1},\ldots ,\alpha_{lk},\ldots ,\alpha_{lK}\right)}^{\prime }$, and $\eta ={\left(\eta_{1},\ldots ,\eta_{L-1}\right)}^{\prime }$ is the structural parameter vector that describes the probability of a randomly selected examinee belonging to the *l*th attribute mastery pattern,

(1) $$p\left(\alpha_{l}|\eta \right)=\frac{\exp\left(\eta_{l}\right)}{\sum_{l=1}^{L}\exp\left(\eta_{l}\right)}$$

note that η_*L*_ is fixed at zero for purposes of model identification (Rupp et al., 2010; Liu et al., 2016a). A *J* × *K* binary Q-matrix $Q=\;(q_{1}{}^{\prime },\ldots ,q_{j}{}^{\prime },\ldots ,q_{J}{}^{\prime }{)}^{\prime }$ specifies the relationships between items and attributes; ${\text{q}}_{j}={\left(q_{j1},\ldots ,q_{jk},\ldots ,q_{jK}\right)}^{\prime }$; *q*_*jk*_ = 1 when the *jth* item requires mastery of the *kth* attribute; and *q*_*jk*_ = 0 otherwise. According to the DINA model, the probability of endorsing item *j* for the *n*th examinee given **α**_*n*_ and **q**_*j*_ is

(2) $$\begin{array}{l} P_{nj}=P\left(x_{nj}=1|{\text{α}}_{n},{\text{q}}_{j}\right)=g_{j}^{\left(1-\gamma_{nj}\right)}{\left(1-s_{j}\right)}^{\gamma_{nj}} \end{array}$$

where $\gamma_{nj}=\prod_{k=1}^{K}{\left(\alpha_{nk}\right)}^{q_{jk}}$ is a binary indicator function, the guessing parameter *g*_*j*_ denotes the probability that an examinee who lacks at least one of the required attributes gives a correct response, and the slipping parameter *s*_*j*_ is the probability that an examinee who has mastered all the required attributes gives an incorrect response.

Although the maximum marginal likelihood with the expectation–maximization (EM) algorithm provides an elegant solution to estimating the model parameters of CDMs, computing the variance-covariance matrix for item parameters is a challenging process under the EM framework. Calculating the matrix requires the inverse of the information matrix in which the item and structural parameters should be simultaneously considered (Liu et al., 2016b). Denote the marginal likelihood of the *n*th examinee's response pattern **x**_*n*_ as

(3) $$\begin{array}{l} L\left(\text{β}|{\text{x}}_{n}\right)=\overset{L}{\underset{l=1}{\sum }}\left[\overset{J}{\underset{j=1}{\prod }}P_{nj}^{x_{nj}}{\left(1-P_{nj}\right)}^{1-x_{nj}}\right]p\left({\text{α}}_{l}|\eta \right) \end{array}$$

where **β** = (**λ**′, **η**′)′ denotes model parameters, $\lambda ={\left(\lambda_{1}^{\prime },\ldots ,\lambda_{j}^{\prime }\ldots ,\lambda_{J}^{\prime }\right)}^{\prime }$ denotes item parameters, and $\lambda_{j}^{\prime }=\left(s_{j},g_{j}\right)$. Then, the log-likelihood function of the observed item response data matrix $x=\text{ (}x_{1}\text{'},\ldots ,x_{n}\text{'},\ldots ,x_{N}\text{' )'}$ is

(4) $$\ell \left(\text{ β}|x\right)=\log L\left(\text{β}|x\right)=\sum_{n=1}^{N}\log L\left(\text{ β}|x_{i}\right)$$

Under the necessary regularity conditions (Bishop et al., 1975), the XPD information matrix is the cross-product of the first-order derivatives of the ℓ(**β**|**x**) with respect to the model parameters **β**:

(5) $$I_{XPD}=\;\left[\frac{\partial \ell \left(\text{ β}|x\right)}{\partial \text{β }}\frac{\partial \ell \left(\text{ β}|x\right)}{\partial \text{β}}\right]$$

The Obs information matrix is the negative of the second-order derivatives of the ℓ(**β**|**x**) with respect to the model parameters **β**:

(6) $$I_{Obs}=-\left[\frac{\ell^{2}\left(\text{β|x}\right)}{\partial \text{β}\partial \beta^{\prime }}\right]$$

Finally, the Sw matrix can be expressed as

(7) $$\begin{array}{l} I_{XPD}=I_{Obs}^{-1}\;I_{XPD}{I_{Obs}}^{-1} \end{array}$$

The detailed derivation process can be found in Liu et al.'s (2018) study. For the DINA model, the XPD and Obs matrices can be expressed as

(8) $$I_{XPD}=\left[\begin{array}{ccc} \frac{\partial \ell \left(\text{β}|x\right)}{\partial g_{1}}\frac{\partial \ell \left(\text{β}|x\right)}{\partial g_{1}} & \cdot \cdot \cdot & \frac{\partial \ell \left(\text{β}|x\right)}{\partial g_{1}}\frac{\partial \ell \left(\text{β}|x\right)}{\partial \eta_{L-1}}\\ \vdots & \ddots & \vdots \\ \frac{\partial \ell \left(\text{β}|x\right)}{\partial \eta_{L-1}}\frac{\partial \ell \left(\text{β}|x\right)}{\partial g_{1}} & \cdot \cdot \cdot & \frac{\partial \ell \left(\text{β}|x\right)}{\partial \eta_{L-1}}\frac{\partial \ell \left(\text{β}|x\right)}{\partial \eta_{L-1}} \end{array}\right]$$

(9) $$I_{Obs}=-\left[\begin{array}{ccc} \frac{\partial^{2}\ell \left(\text{β}|x\right)}{\partial g_{1}\partial g_{1}} & \cdot \cdot \cdot & \frac{\partial^{2}\ell \left(\text{β}|x\right)}{\partial g_{1}\partial \eta_{L-1}}\\ \vdots & \ddots & \vdots \\ \frac{\partial^{2}\ell \left(\text{β}|x\right)}{\partial \eta_{L-1}\partial g_{1}} & \cdot \cdot \cdot & \frac{\partial^{2}\ell \left(\text{β}|x\right)}{\partial \eta_{L-1}\partial \eta_{L-1}} \end{array}\right]$$

### Wald Statistics for DIF Detection

In CDMs, DIF refers to the differences in the probabilities of correctly answering an item for examinees from different groups with the same attribute mastery pattern (Hou et al., 2014; Li and Wang, 2015). Uniform DIF refers to cases when the probabilities of correctly answering an item are uniformly higher or lower for one group across all attribute mastery patterns. Non-uniform DIF occurs if the differences in the probabilities of correctly answering an item between groups depend on the attribute mastery patterns. Theoretically, in the DINA model DIF occurs in item *j* when

(10) $$\begin{array}{l} \Delta_{gj}=g_{\text{F}j}-g_{\text{R}j}\neq 0 \end{array}$$

and/or

$$\begin{array}{l} \Delta_{sj}=s_{\text{R}j}-s_{\text{F}j}\neq \;0 \end{array}$$

where subscript “F” refers to the focal group and “R” refers to the reference group. Item *j* exhibits uniform DIF if Δ_*gj*_ and Δ_*sj*_ have the same signs:

(11) $$\begin{array}{l} \{\begin{array}{c} \Delta_{gj}>0\\ \Delta_{sj}>0 \end{array}\;or\;\{\begin{array}{c} \Delta_{gj}<0\\ \Delta_{sj}<0 \end{array} \end{array}$$

On the other hand, non-uniform DIF occurs.

The Wald test for DIF detection proposed by Hou et al. (2014) in the DINA model evaluates the significance of the joint differences between the item parameters of two groups:

(12) $$\begin{array}{l} W_{d}={\left(\text{C}{\hat{\text{v}}}_{j}\right)}^{\prime }{\left(\text{C}{\hat{\text{Σ}}}_{j}{\text{C}}^{\prime }\right)}^{-1}\left(\text{C}{\hat{\text{v}}}_{j}\right) \end{array}$$

where $v_{j}\text{'}=\left(g_{\text{F}j},s_{\text{F}j},g_{\text{R}j},s_{\text{R}j}\right)$, ${\hat{\text{Σ}}}_{j}$ is the asymptotic variance-covariance matrix associated with the item parameter estimates for both groups, and **C** is a contrast matrix:

(13) $$\text{C}=\left(\begin{array}{cccc} 1 & 0 & -1 & 0\\ 0 & 1 & 0 & -1 \end{array}\right)$$

Under the null hypothesis of *H*_0_: **Cv**_*j*_ **=** **0**, *W*_d_ asymptotically follows a chi-square distribution with 2 degrees of freedom. However, authors of previous studies (Hou et al., 2014; Svetina et al., 2018) showed that *W*_d_ tended to have inflated Type I errors and the *W*_XPD_ and *W*_Obs_ performed better than that for *W*_d_ with regard to Type I error control (Liu et al., 2016b).

### MH and LR

MH and LR are non-CDM-based DIF detection methods. MH evaluates if the examinees' item responses are independent of group membership after conditioning on the observed total score (Mantel and Haenszel, 1959; Mantel, 1963). Let *N*_*m*_ denote the number of examinees with observed total test score *m* from the focal and reference groups. The *N*_*m*_ examinees are cross classified into a 2 × 2 contingency table according to their group membership and their responses to item *j*. Let *A*_*m*_ and *B*_*m*_ denote the numbers of correct and incorrect responses to item *j* in the reference group, respectively. Let *C*_*m*_ and *D*_*m*_ be the corresponding numbers of correct and incorrect responses in the focal group, respectively. The numbers of examinees in the reference group and the focal group are *N*_*m*R_ = *A*_*m*_ + *B*_*m*_ and *N*_*m*F_ = *C*_*m*_ + *D*_*m*_, respectively; the numbers of correct and incorrect responses are *N*_*m*1_ = *A*_*m*_ + *C*_*m*_ and *N*_*m*0_ = *B*_*m*_ + *D*_*m*_, respectively. The MH statistic can be computed by

(14) $$\text{MH}=\frac{{\left\{\left|\sum_{m=1}^{J-1}\left[A_{m}-\text{E}\left(A_{m}\right)\right]\right|-0.5\right\}}^{2}}{\sum_{m=1}^{J-1}\text{Var}\left(A_{m}\right)}$$

where

(15) $$\begin{array}{l} \text{E}\left(A_{m}\right)=\frac{N_{m\text{R}}N_{m1}}{N_{m}} \end{array}$$

and

(16) $$\begin{array}{l} \text{Var}\left(A_{m}\right)=\frac{N_{\text{mR}}N_{m\text{F}}N_{m1}N_{m0}}{N_{m}^{2}\left(N_{m}-1\right)} \end{array}$$

Under the null hypothesis that the examinees' responses are independent of group membership, the MH statistic asymptotically follows a chi-square distribution with 1 degree of freedom.

The LR approach (Swaminathan and Rogers, 1990) is based on the logistic regression model for predicting the probability of a correct response to item *j* from group membership, total test score, and the interaction of these two factors. The full logistic regression model is given by

(17) $$\mathrm{logit}\left(\pi_{n}\right)=\tau_{0}+\tau_{1}M_{n}+\tau_{2}G_{n}+\tau_{3}\left(m_{n}G_{n}\right)$$

where π_*n*_ is the probability that examinee *n* correctly answers item *j*, *M*_*n*_ is examinee *n*'s total score, *G*_*n*_ is the group membership, and τ_0_, τ_1_, τ_2_, and τ_3_ are the regression coefficients. If item *j* does not exhibit any DIF, then τ_2_ = τ_3_ = 0; if item *j* presents uniform DIF, then τ_2_ ≠ 0 and τ_3_ = 0; and if τ_3_ ≠ 0, item *j* shows non-uniform DIF.

## Simulation Design

The purpose of this simulation study was to systematically investigate the Type I error and power performances of *W*_d_, *W*_XPD_, *W*_Obs_, *W*_Sw_, MH, and LR for detecting DIF. The settings of the simulation draw on those of previous real data analyses and simulations on DIF detection methods in CDMs (e.g., de la Torre and Douglas, 2004; Hou et al., 2014; Li and Wang, 2015; Svetina et al., 2018). The test length, sample size, and number of attributes were fixed to *J* = 30, *N* = 1, 000, and *K* = 5, respectively, and the binary item response data sets were generated from the DINA model. The Q-matrix is presented in Table 1.

**Table 1:** Q-Matrix for the simulation study.

**Item**	**Attribute 1**	**Attribute 2**	**Attribute 3**	**Attribute 4**	**Attribute 5**
1	1	0	0	0	0
2	0	1	0	0	0
3	0	0	1	0	0
4	0	0	0	1	0
5	0	0	0	0	1
6	1	1	0	0	0
7	1	0	0	0	1
8	0	1	1	0	0
9	0	0	1	1	0
10	0	0	0	1	1
11	1	1	1	0	0
12	1	1	0	0	1
13	1	0	0	1	1
14	0	1	1	1	0
15	0	0	1	1	1
16	1	0	0	0	0
17	0	1	0	0	0
18	0	0	1	0	0
19	0	0	0	1	0
20	0	0	0	0	1
21	1	0	1	0	0
22	1	0	0	1	0
23	0	1	0	1	0
24	0	1	0	0	1
25	0	0	1	0	1
26	1	1	0	1	0
27	1	0	1	1	0
28	1	0	1	0	1
29	0	1	1	0	1
30	0	1	0	1	1

Five factors that might affect the performance of these methods were manipulated, namely, item quality, attribute correlation, percentage of DIF items, DIF effect size, and DIF type. In the CDM literature (e.g., de la Torre and Douglas, 2004), the guessing and slip parameters of DINA model were typically in the range of (0.1, 0.3), and previous simulation studies (Hou et al., 2014; Liu et al., 2016b) showed that the Type I error rate control of Wald statistics was affected by the item reference slip and guessing parameter values. In this study, the item parameters of the reference group $\lambda_{j}^{\prime }=\left(s_{{\text{R}}_{j}},g_{{\text{R}}_{j}}\right)$ for the high, medium, and low item quality conditions were fixed to 0.1, 0.2, and 0.3, respectively. In previous DIF simulation studies (e.g., Hou et al., 2014; Liu et al., 2016b) the correlation coefficient between two attributes was fixed to 0, however, according to Kunina-Habenicht et al. (2012), attribute correlation coefficient was typically in the range of (0.5, 0.8). In this study, three attribute correlation coefficient levels ρ = 0, 0.5, and 0.8 were considered, which allowed for a more realistic depiction of the attribute correlation between attributes seen in practical cognitive diagnostic assessments. The percentage of DIF items had two levels, 10 and 30%. The DIF effect size had two levels, 0.05 (small DIF) or 0.1 (large DIF). There were also two DIF types, uniform or non-uniform. The summary of DIF conditions are shown in Table 2. Note that to ensure that the item parameters for the focal group would be larger than zero, for the **λ**_*j*_ = 0.1 condition, only the small DIF size was considered. This yielded 240 conditions for data generation. For each simulation condition, 200 converged replications were used to evaluate the performance of DIF detection methods. The simulation study was implemented in R (R Core Team, 2018), the R packages *CDM* (Robitzsch et al., 2018) and *dcminfo* (Liu and Xin, 2017) were used to estimate the model parameters and the asymptotic covariance matrices of item parameter estimates, respectively, the R functions for Wald statistic calculation were modified from the *CDM* package, the MH and LR tests were performed using the R package *difR* (Magis et al., 2010). The R codes in this study are available upon request from the corresponding author.

**Table 2:** Summary of DIF conditions for the simulation study.

**DIF Type**	**DIF size**	****Δ**_*gj*_ = *g*_*Fj*_−g_Rj_**	****Δ**_*sj*_ = *s*_*Rj*_−*s*_*Fj*_**
Uniform	0.05	+	+
		–	–
	0.1	+	+
		–	–
Non-uniform	0.05	+	–
		–	+
		+	0
		0	+
		–	0
		0	–
	0.1	+	–
		–	+
		+	0
		0	+
		–	0
		0	–

For the purpose of this study, the performance of the *W*_d_, *W*_XPD_, *W*_Obs_, *W*_Sw_, MH, and LR methods was evaluated in terms of Type I error rate and power. Type I error rate was computed as the proportion of non-DIF items incorrectly flagged as DIF items. On the other hand, empirical power was computed as the proportion of DIF items that were correctly identified. The empirical Type I error rate of the DIF detection method in the interval [0.025, 0.075] for the nominal level of 0.05 was considered to be accurate (Bradley, 1978).

## Results

**Figure 1:**
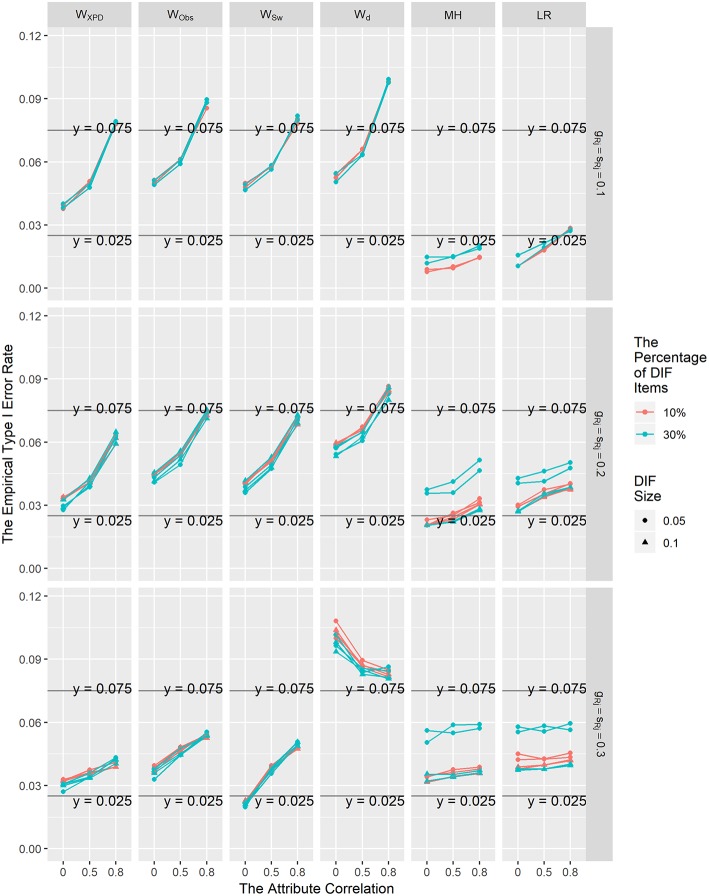
The Type I error rates for the *W*_d_, *W*_XPD_, *W*_Obs_, *W*_Sw_, MH, and LR methods under the uniform DIF condition.

The averaged Type I error rate control results for these six methods under the uniform and non-uniform DIF conditions for different percentages of DIF items, attribute correlations, and reference item parameters across the 200 replications showed similar patterns; thus, only the Type I error rate results for the uniform DIF condition are shown graphically in Figure 1. In general, the empirical Type I error rates for *W*_XPD_, *W*_Obs_, and *W*_Sw_ were better than those for *W*_d_ under all conditions. Consistent with the results reported in previous studies (e.g., Hou et al., 2014; Wang et al., 2014; Svetina et al., 2018), the Type I error rates for *W*_d_ were somewhat inflated under most of the conditions. Moreover, although the performances of *W*_XPD_, *W*_Obs_, and *W*_Sw_ seemed to be influenced by the attribute correlation, the Type I error rates for those methods were reasonably close to the nominal Type I rate of 0.05 under most of the simulation conditions. For most conditions, *W*_XPD_ had good performance in controlling Type I error rates; the only exceptions were for conditions ρ = 0.8 and **λ**_R*j*_ **=** 0.1, for which the Type I error rates were slightly higher than the nominal level. The Type I error rates were close to the nominal level for *W*_Obs_ and *W*_Sw_ on most occasions except when **λ**_R*j*_ **=** 0.1, or **λ**_R*j*_ **=** 0.2 under the ρ = 0.8 condition. The performance of the *W*_XPD_ was found to perform slightly better than *W*_Obs_ and *W*_Sw_ in controlling Type I error rates when ρ = 0.8. It was found that under the null hypothesis, MH and LR tended to be somewhat conservative, with Type I error rates consistently below the nominal level when **λ**_R*j*_ **=** 0.1. The Type I error rates for *W*_XPD_ and LR were in the range of [0.025, 0.075] under most of the simulation conditions, which suggested that *W*_XPD_ and LR had the best performance in controlling Type I error rates among the six methods investigated in this study.

**Figure 2:**
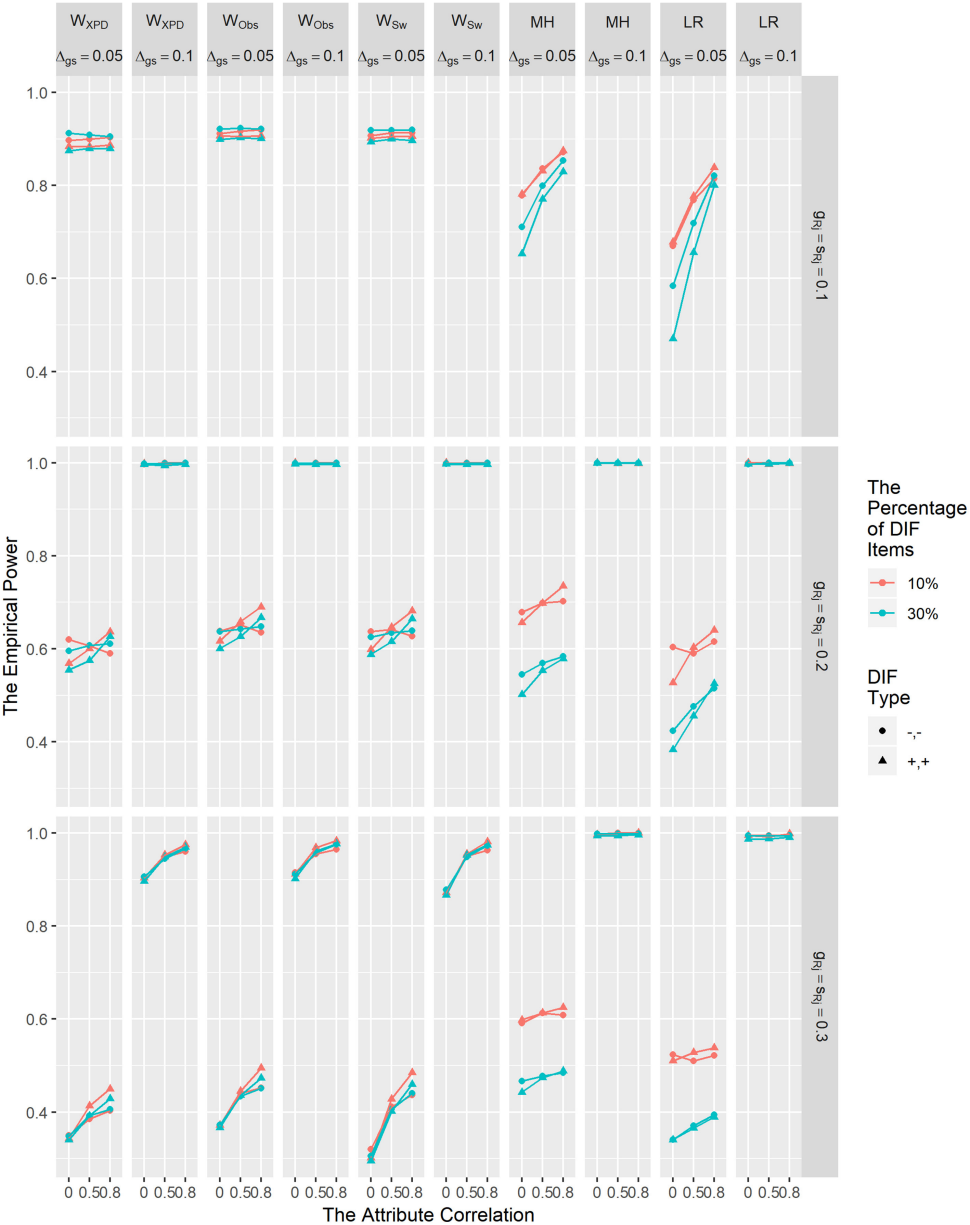
The empirical power results of the *W*_XPD_, *W*_Obs_, *W*_Sw_, MH, and LR methods under the uniform DIF condition.

The empirical power results for *W*_XPD_, *W*_Obs_, *W*_Sw_, MH, and LR for detecting uniform DIF are shown in Figure 2. The power results for *W*_d_ method are not reported, due to its inflated Type I error rates. In Figure 2, it is clear that the DIF size and reference item parameter values influenced the power rates of the *W*_XPD_, *W*_Obs_, *W*_Sw_, MH, and LR; as the DIF effect size increased, the power rates of these five methods increased. Specifically, when DIF size was 0.1, the power rates were all above 0.8, and when DIF size was 0.05, the power of these methods decreased as item parameter values increased. The power for *W*_XPD_, *W*_Obs_, and *W*_Sw_ was comparable with or superior to that for MH and LR under **λ**_R*j*_ **=** 0.1 and **λ**_R*j*_ **=** 0.2 condition; in contrast, *W*_XPD_, *W*_Obs_, and *W*_Sw_ were less powerful than MH and LR under **λ**_R*j*_ **=** 0.3. The power for *W*_XPD_, *W*_Obs_, and *W*_Sw_ increased as attribute correlation values increased when DIF size was .05. Figure 2 demonstrates that for the MH and LR methods, when DIF size was 0.05, the power increased as the proportion of DIF items decreased or the attribute correlation increased. In contrast, for *W*_XPD_, *W*_Obs_, and *W*_Sw_, the power decreased as the attribute correlation increased when DIF size was 0.05 and **λ**_R*j*_ **=** 0.3. Similar to the results reported by Hou et al. (2014), we found that the power for *W*_XPD_, *W*_Obs_, and *W*_Sw_ was not affected by the percentage of DIF items.

**Figure 3:**
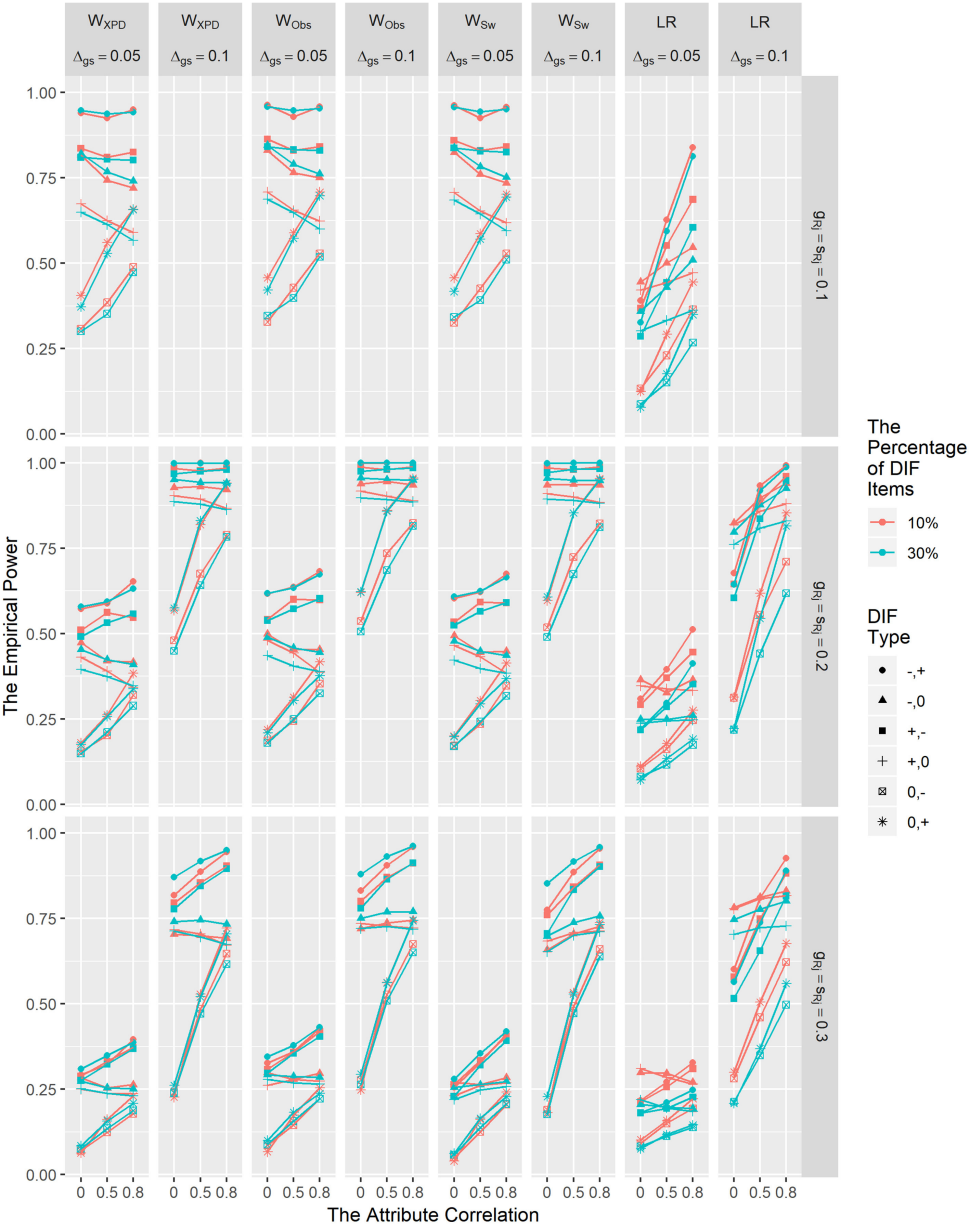
The empirical power results of the *W*_XPD_, *W*_Obs_, *W*_Sw_, and LR methods under the non-uniform DIF condition.

Figure 3 depicts the power results for *W*_XPD_, *W*_Obs_, *W*_Sw_, and LR for detecting non-uniform DIF. The power results for MH are not presented in Figure 3, since MH is only capable of detecting uniform DIF. In general, the power of *W*_XPD_, *W*_Obs_, and *W*_Sw_ was comparable with or higher than that of LR under all conditions. As shown in Figure 3, the power rates for the non-uniform DIF conditions were similar to those for the uniform DIF conditions in that the power increased with larger DIF size, and smaller reference item parameter values regardless of other factors. Close inspection of the results in Figures 2, 3 reveals that the power of the MH and LR methods decreased with more DIF items under most of the conditions; in contrast, the power of the Wald statistics was not affected by the percentage of DIF items.

## Summary and Discussion

Given the fact that detecting and eliminating DIF items from cognitive diagnostic tests is important for test fairness and validity, researchers have proposed a number of CDM-based and non-CDM-based DIF detection methods. Previous studies (Hou et al., 2014; Svetina et al., 2018) found that although the power of *W*_d_ was comparable with or better than that of LR and MH, the Type I error rate for *W*_d_ can be inflated under certain conditions because of the method's underestimated item parameter covariance matrix. Alternative information matrix estimation methods such as XPD, Obs, and Sw have been proposed to calculate item parameter covariance matrices in CDMs in which the item parameters and structural model parameters are simultaneously considered (Liu et al., 2016b, 2018; Philipp et al., 2018). Motivated by these findings, in the current study we sought to systematically evaluate the performance of the Wald tests based on item-wise, XPD, Obs, and Sw matrices and to compare the behavior of the CDM-based DIF detection methods *W*_d_, *W*_XPD_, *W*_Obs_, and *W*_Sw_ and the non-CDM-based DIF methods MH and LR under various simulation conditions.

In this study, it was found that the Type I error rate control of *W*_XPD_, *W*_Obs_, and *W*_Sw_ was generally better than that of *W*_d_. *W*_XPD_ had slightly better performance in controlling Type I error rates than did *W*_Obs_ or *W*_Sw_ under most conditions. The power of *W*_XPD_, *W*_Obs_, and *W*_Sw_ was generally better than that for LR and MH, especially when the item quality was medium or high under most of the simulation conditions. As far as we are aware, this study is the first to compare the Wald statistic based on the XPD, Obs, or Sw matrix with LR and MH. The results provide strong evidence that among the six DIF detection methods investigated in this study, *W*_XPD_ performed best in terms of Type I errors and power under most of the conditions. We believe the current study contributes valuable information regarding the DIF detection methods in CDMs for practical implications.

In spite of the encouraging results, there are, of course, a number of limitations that should be noted here. First, it should be noted that in CDM-based DIF detection, the good performance of the Wald statistic depends highly on the accuracy of the item parameter estimates; for example the Type I error for *W*_XPD_ was somewhat high when **λ**_R*j*_ **=** 0.1 and ρ = 0.8, and its power decreased as the item quality decreased.

Second, the test length, sample size, and number of attributes in this study were fixed. To further generalize the simulation results, future studies that involve a wider range of conditions are needed. Third, even though this simulation was carefully designed to mimic those of CDM practices and simulations on DIF detection methods (de la Torre and Douglas, 2004; Hou et al., 2014; Li and Wang, 2015; Svetina et al., 2018), the findings of this study rely on the assumptions that the fitted model and the corresponding Q-matrix were correctly specified, which may limit the generalizability of our findings. For example, in the present study, the DINA model, which has been frequently used in applications, was taken as an example to compare the performance of the DIF detection methods. However, DINA is one of, if not the most restrictive, simplest model, and previous studies (Ma et al., 2016; Liu et al., 2018) have shown that no single CDM suits all the test items in many, if not all, diagnostic applications. It would be useful to examine the empirical behavior of the DIF detection methods in the in the context of general CDMs, such as the general diagnostic model (von Davier, 2008), the log-linear cognitive diagnosis model (Henson et al., 2009) and the generalized DINA model (de la Torre, 2011). Fourth, model misspecification is virtually unavoidable in real-world data analyses (Liu et al., 2016a), further studies are needed to investigate the performance of the DIF detection methods under varying degree of misspecified models, especially with real data examples. Since we believe more simulations are needed before researchers can be sure how to use DIF detection methods safely in CDMs, real data analyses were not conducted in this study.

In conclusion, the simulation study shows that *W*_XPD_, *W*_Obs_, and *W*_Sw_ perform better than *W*_d_, LR, and MH in terms of Type I errors and power.

## Author Contributions

YL and TX developed the original idea, carried out the simulations and analyses. YL, HY, LS, and LY were involved in drafting and revising the manuscript. All authors approve the final manuscript submitted.

### Conflict of Interest Statement

The authors declare that the research was conducted in the absence of any commercial or financial relationships that could be construed as a potential conflict of interest.
